# Unusual course of disease and genetic profile in Li-Fraumeni syndrome-associated osteosarcoma – a case report

**DOI:** 10.1186/s13053-021-00202-0

**Published:** 2021-10-20

**Authors:** Alexander Puzik, Markus Uhl, Juri Ruf, Tilmann Schumacher, Udo Kontny

**Affiliations:** 1 Department of Pediatric Hematology and Oncology, Medical Center – University of Freiburg, Faculty of Medicine, University of Freiburg, Mathildenstrasse 1, D-79106 Freiburg, Germany; 2 Division of Pediatric Radiology, Department of Radiology, Medical Center – University of Freiburg, Faculty of Medicine, University of Freiburg, Mathildenstrasse 1, D-79106 Freiburg, Germany; 3 Department of Nuclear Medicine, Medical Center – University of Freiburg, Faculty of Medicine, University of Freiburg, Hugstetter Strasse 55, D-79106 Freiburg, Germany; 4 Practice for Nuclear Medicine, Wirthstr. 11, D-79110 Freiburg, Germany; 5 Department of Pediatric Hematology, Oncology and Stem Cell Transplantation, RWTH Aachen, Pauwelsstrasse 30, D-52074 Aachen, Germany

**Keywords:** Osteosarcoma, Li-Fraumeni syndrome, Genetics, Copy number alterations

## Abstract

**Background:**

Osteosarcoma is a highly malignant tumour associated with numerous and complex genetic alterations like copy number alterations. Recent whole genome studies revealed distinct mutations in several candidate oncogenes.

While clinical parameters stratify osteosarcoma patients in risk groups, genetic profiles have not yet been used to tailor tumour treatment. However, specific copy number alterations seem to have a prognostic impact in osteosarcoma treatment.

Somatic *TP53* gene mutation frequently occurs in sporadic osteosarcoma. When arising germline, *TP53* mutation leads to Li-Fraumeni syndrome and may result in early life osteosarcoma. The effect of Li-Fraumeni syndrome on the genetic profile of osteosarcoma and the consideration of the syndrome during cancer treatment are topics of current research.

**Case presentation:**

We report a 25-year-old female with pelvic osteosarcoma refusing continuation of therapy. She interrupted neo-adjuvant chemotherapy according to EURAMOS-1/COSS recommendations and declined local or further adjuvant therapy. Surprisingly, she remained in sustained remission for the osteosarcoma but eventually died from newly diagnosed breast cancer. After establishment of breast cancer, we detected *TP53* germline mutation and investigated the osteosarcoma material with array-CGH.

**Conclusion:**

Genetic examination of the tumour evidenced several copy number alterations with striking differences to previously reported data. We discuss possible influences of the genetic profile on the unusual clinical course and the significance of Li-Fraumeni syndrome for the genetic profile. Specific loss of (proto-) oncogenes might have contributed to the unusual case. Further large-scale genetics of Li-Fraumeni patients combined with detailed clinical data will help to identify specific genetic risk profiles and improve treatment.

**Supplementary Information:**

The online version contains supplementary material available at 10.1186/s13053-021-00202-0.

## Background

Osteosarcoma (OS) is a high-grade malignant bone-producing sarcoma and represents the most common malignant skeletal tumour of young adults with an incidence of 4/1.000.000. Prognosis is generally favourable, but depends on several risk factors. Tumour localisation and local therapy, presence of metastasis and chemotherapy response are the most relevant prognostic factors [1]. Without local therapy the survival rates decline to approx. 10 to 20% [2]. Several research groups reported the impact of distinct sets of copy number alterations (CNA) and other genetic changes on prognosis. Smida et al. (2010) were able to predict treatment response better by genetic changes like CNA compared to the Salzer Kuntschik grading. Yen et al. (2009) identified CNA specific for recurrent or metastatic OS [3, 4]. However, OS study groups worldwide have not introduced stratification into genetic risk groups into standard care.

Upon genetic analysis complex karyotypes with multiple numerical and structural chromosomal alterations originating from a process called chromothripsis are very frequent in OS compared to other cancers [1, 5]. OS is one of the tumours with the highest rate of CNA and mutations [6]. Genomic instability even results in genetically diverse cell populations within a single OS. Specific CNA like deletions of pro-apoptotic genes seem to induce selection advantages and work as biological filters for other “transient” CNA [5].

Somatic mutations found in OS and other cancers very frequently affect *TP53*, the prototype of tumour suppressor genes. Young female adults are frequently diagnosed with *TP53*-mutated OS and *TP53* mutation frequently occurs in very young sarcoma patients [7]. Moreover, germline *TP53*-mutation leading to Li Fraumeni-syndrome (LFS) is one of the major predispositions for sarcoma development [7, 8]. Whole genome studies of OS have identified several other somatic mutations in candidate oncogenes [9].

LFS is an autosomal-dominant inherited tumour predisposition syndrome leading to multiple, specific cancers and a determinately increased lifetime risk for cancer reflected in the current LFS or Chompret criteria [8, 10]. Early-onset breast cancer (age 30–40) is the leading LFS cancer. Germline *TP53* mutation is thought to be responsible for the initiation of oncogenesis due to the gene’s numerous functions in response to stress, cell cycle control, apoptosis and DNA damage repair [8, 10].

Interestingly, LFS patients demonstrated shorter telomeres and increased and complex CNA reflecting germline genomic instability [8, 11]. Array-CGH analyses of *TP53*-negative LFS families and controls have not detected any changes in CNA [11].

There is no data demonstrating specific or redundant sets of CNA in LFS patients associated with a certain profile of tumours. Regarding OS, *TP53*-mutated OS usually seems to harbour less mutations than *TP53*-wild type OS [6]. However, further data evaluating the differences between genetic profile of sporadic OS and LFS-associated OS is missing.

## Case presentation

A 25-year-old female professional dancer presented to the sports medicine department due to increasing pain in the left iliac crest with radiation to the left leg. She initially noticed the pain after chiropractic treatment 6 months before (see timeline in Additional file 1). Further medical history of the patient was unremarkable. After the clinical diagnosis of lumbar syndrome was established, the sports physician prescribed acupuncture and physiotherapy. Both did not ease the pain. Next, a swelling of the left hip region appeared. Ultrasound showed no signs of arthritis but raised suspicion of a soft tissue mass. The patient was then referred to the adult oncology department.

**Fig. 1:**
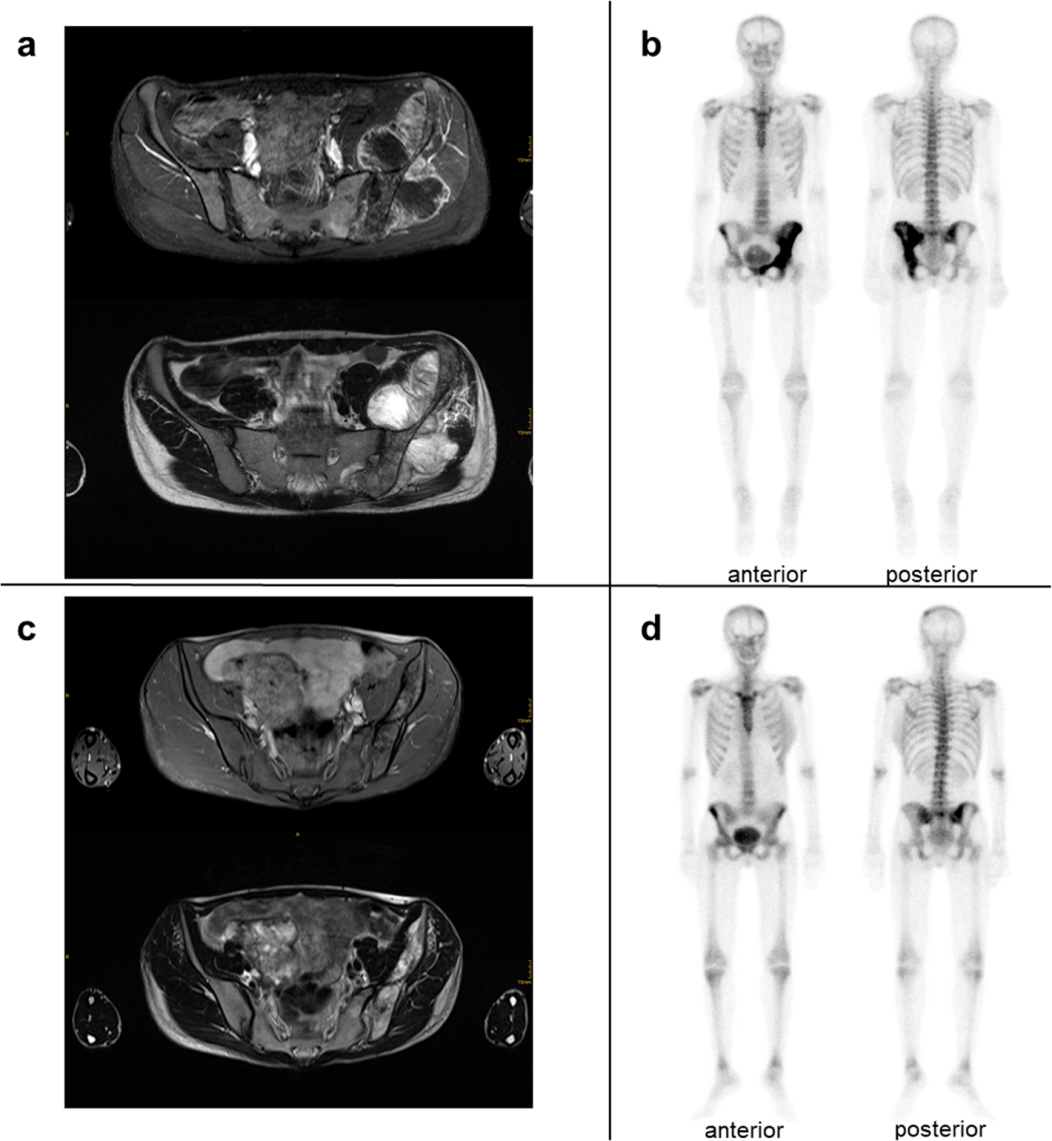
Imaging results of patient at diagnosis (a/b) and last follow-up 26 months later (c/d). **a** Axial MRIs of the pelvis showing chrondroblastic osteosarcoma of the left Os ileum with infiltration of gluteal muscles, left iliac and obturatorius internus muscle, periosteous soft tissue, Os sacrum and Os pubis with strong contrast enrichment. T_1_-weighted transversal MRI with i.v.-contrast-medium (gadoteriol, Prohance®, Bracco) and fat saturation (above) and T_2_-weighted transversal MRI (bottom). **b** Aside from intensive bone turn over at the left hemipelvis, no other suspicious bone lesions are observed in initial bone scan. **c** Axial MRIs of the pelvis showing residual proportions of chrondroblastic osteosarcoma of the left Os ileum with decreasing contrast enrichment and signs of sclerosis and ossification of small soft tissue component. T_1_-weighted MRI with contrast agent and fat saturation (above) and T_2_-weighted MRI (bottom). **d** Bone scan demonstrating a normalisation of bone turn over at the left hemipelvis. MRI could rule out an initially suspected left parietal metastasis. Aside from an accentuation of the right sacroiliac joint due to incorrect weight bearing, no other suspicious bone lesions were observed

Magnetic resonance imaging (MRI) of the pelvis revealed a large tumour of the left iliac bone with infiltration of periosteal muscles and soft tissue. Left-sided pubic bone, sacrum and sacroiliac joint were infiltrated (Fig. 1a). Skeletal scintigraphy revealed no further osseous lesions (Fig. 1b), computed tomography (CT) of chest and abdomen excluded metastases. A biopsy was performed, which resulted in diagnosis of OS, grade III of chondroblastic subtype. Despite recommendation for chemotherapy, the patient decided to perform a fasting cure and lost 6 kg weight.

A further MRI was done a couple of weeks later due to increasing, intolerable pain, despite treatment with opioids, cox-2-inhibitors, non-steroidal anti-inflammatory drugs and coanalgesics. Tumour size remained stable. The patient then decided to start chemotherapy according to recommendations of EURAMOS-1/COSS protocol in the paediatric oncology department.

Under chemotherapy the pain subsided within 2 months and the tumour size decreased without relevant side effects except for nausea. Due to her profession as a dancer, the patient refused to undergo local therapy by surgery or radiation and discontinued chemotherapy after 4 months. We discussed the very poor prognosis of OS without local therapy in detail with the patient.

Much to our surprise, we found no signs of tumour relapse or metastases within the next year. Imaging studies displayed a stable residual tumour mass of the left iliac bone with further decline of contrast enhancement and soft tissue portions (Fig. 1c).

Unfortunately, 16 months after the end of chemotherapy the patient first recognized a lump in her left breast. Clinical examination revealed enlarged lymph nodes (2 cm) of the left axilla. The patient refused mammography; ultrasound indicated possible breast cancer. A couple of weeks later, ultrasound-guided biopsy of the breast lump was performed resulting in the diagnosis of invasive ductal carcinoma (G2, B5b, ER/PR 0%, HER2/neu score 3, MIB-1 30%). Again, the patient declined biopsy of the lymph nodes. MRI of the chest revealed destructive carcinoma of the complete left mamma without signs of pulmonary metastases (TNM: cT2, cN1, cM0). Skeletal scintigraphy neither displayed activity of the pelvic OS nor of other bone lesions. Head MRI ruled out an initially suspected parietal metastasis with slightly increased tracer uptake (Fig. 1d). Ultrasound of the abdomen displayed a hypo-echogenic nodule of the liver.

Once again the patient refused any further treatment. Two months later, she was admitted to the gynaecologic department with severe headaches and intermittent visual field loss. The gynaecologists interpreted findings as migraine headaches due to spontaneous improvement of pain and unremarkable MRI of the head. A couple of days later, she presented to the emergency department with sudden deterioration of her general condition, dyspnoea and decreased oxygen saturation. A CT of chest and abdomen revealed diffuse pulmonary consolidations, pericardial effusion and several hypodense hepatic nodules consistent with disseminated end-stage breast cancer. Upon further deterioration, the patient and her family agreed to withhold invasive procedures and the patient soon died of respiratory failure.

After establishing the diagnosis of breast cancer, we performed genetics for Li-Fraumeni syndrome and confirmed the diagnosis (common *TP53* mutation: DNA binding domain, c.733G > A, p.Gly254Ser, heterozygous). Family history of the patient was unremarkable regarding classical Li-Fraumeni criteria, the patient’s father died from a pulmonary tumour at old age.

**Fig. 2:**
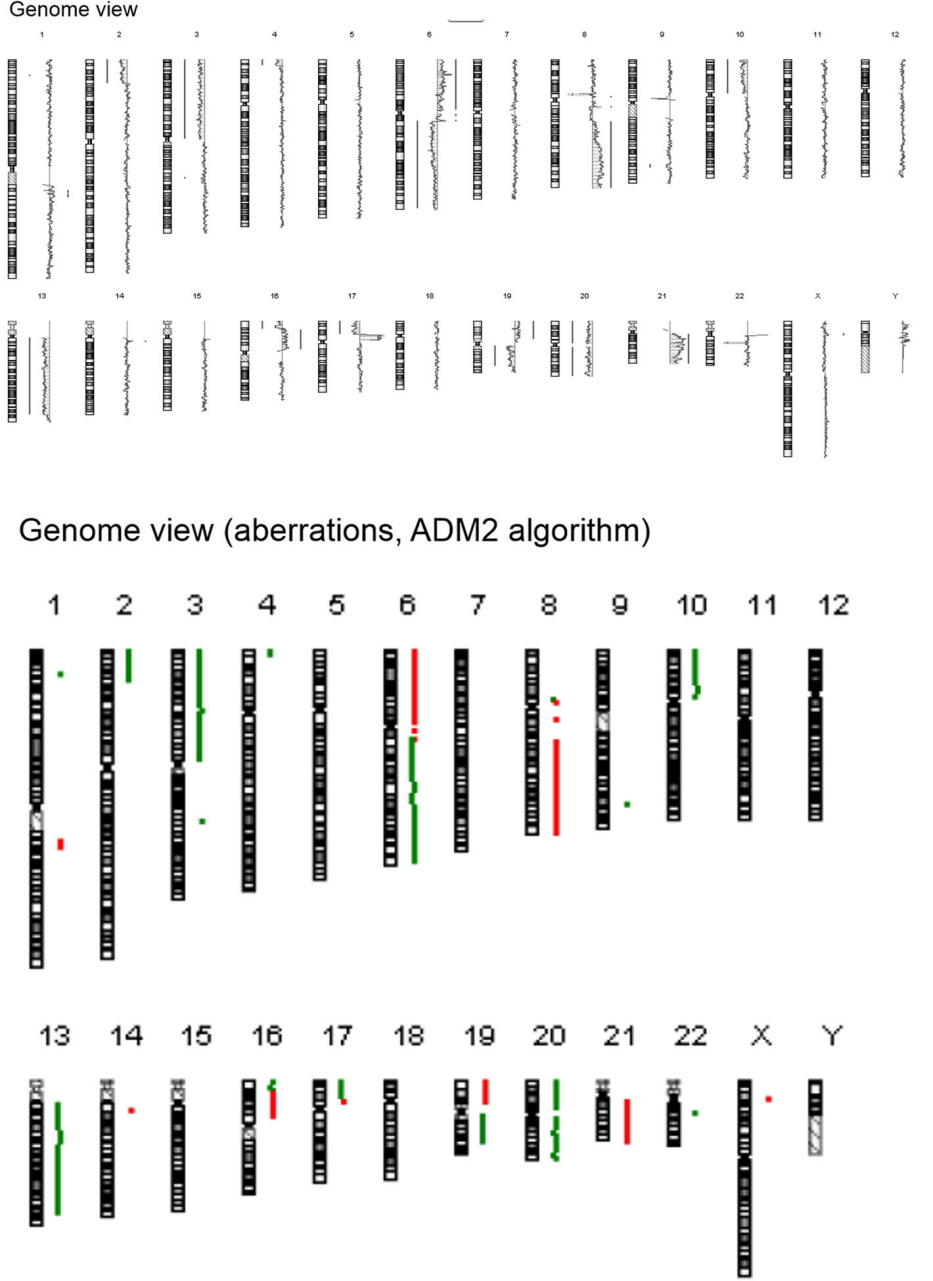
Results of array-CGH. Genome view and view of aberrations (ADM2 algorithm). Copy number losses are depicted in green colour, copy number gains in red colour

To elucidate the very uncommon course of the OS without signs of vital tumour, despite absence of local therapy and incomplete chemotherapy, we performed array-comparative genomic hybridization (aCGH) of the tumour material according to routine protocols (Fig. 2). Table 1 compares common CNA in OS with the individual CNA of our LFS patient.

**Table 1:** Comparison of frequently reported CNA of osteosarcoma with individual CNA of LFS patient

Chromosome	CNA of current OS		Frequent CNA in OS	
	Chromosomal regions			
	gain	loss	gain	loss
1	*1q23*	**1p36**	**1p32–36**, *1q21–31*	1q25.1
2		2p23.2–25.2		2q
3		*3p*, 3q22.3	3q26	*3p14.1,* 3q13
4		4p16.2–3		4p15.1
5			**5p13-cen**	
6	*6p-cen*	*6cen-q*	*6p12–21*	6p, *6q12,* *6q16, 6q21–22*
7				
8	8q21–24.3	**8p11.22**	**8p**, 8cen-q13, *8q12–24*	8p21
9		9q33.1		9p13
10		*10p*		*10p,* 10q22–26
11			11q14	
12			12p13–14, 12q11–14	
13		*13q*		*13q12.2, 13q14,* *13q21–31*
14	14q11.2		14q24	
15				
16	*16p12.1–13.2*	**16p13.2–3**	*16p13*	
17	*17p11.2*	**17p12–13**	*17p11***-17p13**, 17q21–25	*17p13,* 17q12
18				18q21–23
19	*19p*	**19q**	*19p13*, **19q12–13, 19q**	
20		20p, **20q**	**20q**	
21	21q			
22		22q11.2		
X,Y	Xp22.11		Xp11.2–21, Xq12	

Frequently reported CNA refer to data of Rosenberg et al., Sadikovic et al., Overholtzer et al. and Man et al. Similar CNA were depicted in italics, clearly opposing CNA in fat letters. Most frequently reported CNA were underlined.

## Discussion and conclusions

OS patients without adequate local therapy display dismal survival rates with high chance of relapse and metastasis [2]. Local therapy next to chemotherapy response (and absence of metastasis) is one of the main predictors of the survival of OS patients [1, 12]. Persistent remission of OS as confirmed by lack of contrast enrichment and unremarkable diffusion parameters in MRI (Fig. 1c) after incomplete chemotherapy without local therapy is very unusual. Moreover, the patient’s *TP53* mutation is usually known to be associated with an unfavourable course of OS [7, 10].

We elucidate below the differences in genetic profile between available data and our LFS-associated OS to raise the question as to whether a distinct genetic profile provides explanations for the unusual clinical course.

In general, we detected numerous CNA comparable to pooled data of Overholtzer et al. (2013), Rosenberg et al. (2013), Sadikovic et al. (2003) and Man et al. (2004) [1, 5, 13, 14]. Particularly, the tumour evidenced much less copy number gains than previously reported in sporadic OS. Further, we were able to detect 25 independent chromosomal aberrations (especially losses) compared to the reported average count of independent chromosomal lesions around ten. Chromosomes 5, 7, 11, 12, 15 and 18 displayed no changes, chromosomes 1, 4, 9, 14, 22 and X had just minor CNA. Data regarding CNA in LFS-associated OS is not available.

Compared to the eleven most frequent aberrations out of 65 OS reported by Overholtzer et al. (2003) the current tumour evidenced five of these (+8q24, +6p12, +17p11.2, −6q16, −10p12pter) [13]. Expanded to 28 frequent aberrations including data of Sadikovic et al. (2013) and Rosenberg et al. (2013) the current tumour demonstrated five out of 16 frequent copy number increases and five out of 12 frequent copy number losses [1, 5]. Compared to the CNA reported by Smida et al. (2010), which were predictors of poor treatment response in OS, the current tumour evidenced only one of these (+8q24), whereas the others occurred opposing (e.g. gain of 6q21 instead of reported loss) or were not present (+12q14). Furthermore, these authors found poor treatment response, when more than six chromosomes were affected, especially chromosomes 2, 3, 5, 6, 10 and 13 [3]. This data opposes the favourable course of our highly altered OS with 16 chromosomes affected by CNA. Compared to the CNA associated with recurrence and metastasis reported by Yen et al. (2009) the current tumour evidenced three of these (−6q14.1, −6q16.2–22.31, +8q22.3–24.3), whereas the others were not detectable (−8p23.2–12, +8q21.12) or opposing (+17p12) [4]. We present a detailed comparison of frequently reported CNA in sporadic OS and CNA of our case in Table 1.

As a result of this strikingly different distribution of CNA compared to the available data, we wondered about subsequent effects on candidate oncogenes. Sadikovic et al. (2009) postulated the association between certain CNA and oncogenesis in OS discussing oncogenic effects of the amplification of the *RUNX2* gene at 6p12.3–21.1 among others [15]. Those genes known to play a role in oncogenesis and possibly affected by CNA in our case are described in the Atlas of Genetics and Cytogenetics in Oncology and Hematology and overviewed in Additional file 2 [16].

In the context of the highly complex genetic structure of OS the following genetic alterations received further attention.

We found loss of 1p36 instead of the high-frequent gain of 1p32–36 in previous reports [1, 5, 13]. The chromosomal region harbours several genes associated with migration, proliferation and adhesion, e.g. *YBX-1* and *ARID1A*. *YBX-1* (involved in splicing, transcriptional regulation, translation, chromatin remodelling and DNA repair) was usually overexpressed in OS and associated with a higher proliferative index [17]. *ARID1A* is a chromatine modifier and known oncogene in OS [9].

Loss of 20q and 19q has rarely been reported in OS in contrast to high-frequent gains of these regions [1, 5, 13]. Both regions comprise several candidate proto-oncogenes (20q: NKX2.2, PCNA, PLCB1, *DNMT3B, E2F1, SRC, TPX2, CHD6, PLCG1, EYA2, MYBL2, UBE2L, CSE1L, SNAI1, AVRKA, TNFRSFCB, PTK6; 19q****:*** *FXYD3, FXYD5, ACTN4, AKT2, AXL, CEACAM1, CEACAM5, CIC, CXCL17, PAF1, TGFB1, CADM4, LYPD3, PLAUR, BBC3, MARK4, RELB, SLC1A5, AKT1S1, ATF5, BAX, BCL2L12, CARD8, EMP, MIR150, RUVBL2***)** as well as some oncogenes already known to have tumour promoting effects in OS (20q: *BLCAP, GNAS*; 19q: *AKT2*) [18, 19].

Next, we discuss the impact of LFS on genetic profile of OS and outcome. To the best of our knowledge, studies comparing genetic profile and outcome between LFS-associated OS and sporadic OS are not available. LFS itself is associated with genomic instability determined by *TP53* mutation. The mutation of *TP53* has certainly laid the foundation for development of random genomic alterations in subsequent tumours and is an important, but not mandatory part of OS development [5, 7, 8, 11]. Clearly conflicting with our data, Bousquet et al. (2016) reported fewer mutations in *TP53*-mutated OS (without germline *TP53*-mutation) than in wild type OS [6]. Therefore, one might ask whether different time points and origins of *TP53* mutation (germline vs. somatic) have an impact on the evolution of genetic profile of OS and other typical LFS tumours. Finally, even if all of the above-mentioned alterations have arisen by chance, such a genetic profile might simply be specific for LFS-associated OS. The influence of genetic profiles of OS on prognosis has already been reported [3, 4, 15].

In conclusion, we report on a very unusual course of OS in a patient with Li-Fraumeni syndrome remaining in persistent remission after incomplete chemotherapy and without local therapy. Furthermore, we tried to specify differences between CNA in reported OS cases and our case and asked about a potential role of LFS for our findings. In our case, certain losses of (proto-) oncogenes might have played a major role in disease progression. Variations in therapy response, sensitivity to therapy and outcome are well known during treatment of cancers of other tumour predisposition syndromes like trisomy 21 or ataxia teleangiectasia.

International analysis of combined genetics and clinical data of LFS studies like the Clinical and Genetic Studies of Li-Fraumeni Syndrome of the US National Cancer Institute (NCT01443468) or the Li-Fraumeni-Syndrome-Cancer-Predisposition-Syndrome Registry 01 (LFS-CPS-R01) of the German Society of Pediatric Hematology and Oncology (GPOH) [20] will result in the identification of a specific genetic profile of LFS tumours. These genetic profiles will improve stratification of therapy and the outcome.

## Supplementary Information

**Additional file 1.** Patient’s timeline.

**Additional file 2.** Detailed list of potentially relevant genes affected by different CNA profile. List of potentially affected genes due to CNA and potentially affected genes due to differences to previously reported CNA. Validated (candidate) oncogenes in OS according to Rickel et al. (2017) were depicted in fat letters.

## Data Availability

Data sharing is not applicable to this article as no datasets were generated or analysed during the current study.

## Abbreviations

aCGH: Array-comparative genomic hybridization
CNA: Copy number alterations
CT: Computed tomography
LFS: Li-Fraumeni syndrome
MRI: Magnetic resonance imaging
OS: Osteosarcoma
