# Supplementary figures and images for: Unusual course of disease and genetic profile in Li-Fraumeni syndrome-associated osteosarcoma – a case report
Source: Hered Cancer Clin Pract. 2021 Oct 20;19:44. doi: 10.1186/s13053-021-00202-0 (PMC8527636; doi:10.1186/s13053-021-00202-0)

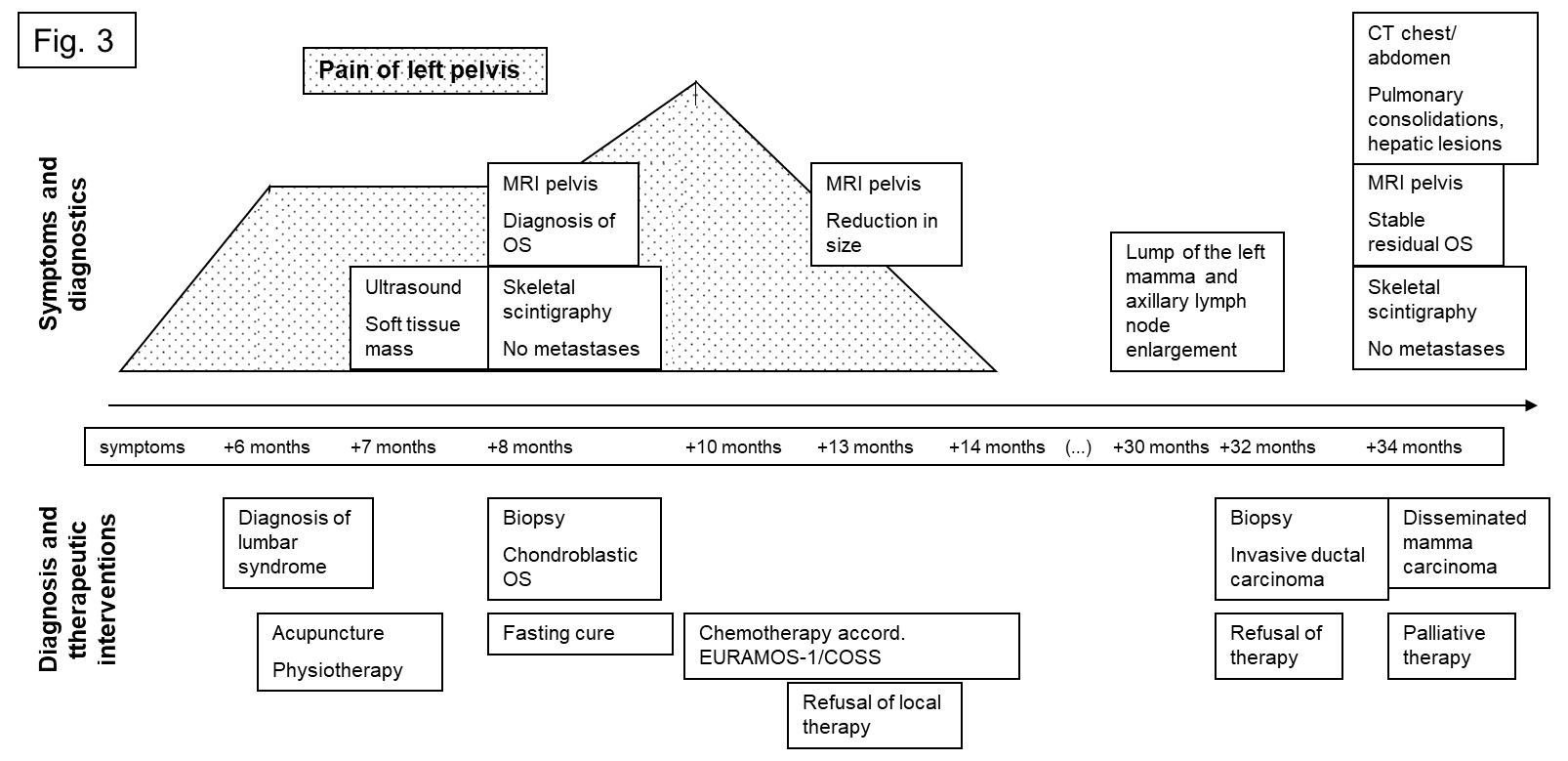

Supplement: Supplementary file 1 — Additional file 1. Patient’s timeline. [file 13053_2021_202_MOESM1_ESM.png]
